# Harnessing Endophytic Fungi for Enhancing Growth, Tolerance and Quality of Rose-Scented Geranium (*Pelargonium graveolens* (L’Hér) Thunb.) Plants under Cadmium Stress: A Biochemical Study

**DOI:** 10.3390/jof7121039

**Published:** 2021-12-03

**Authors:** Nadia Mohamed El-Shafey, Marym A. Marzouk, Manal M. Yasser, Salwa A. Shaban, Gerrit T.S. Beemster, Hamada AbdElgawad

**Affiliations:** 1 Department of Botany and Microbiology, Faculty of Science, Beni-Suef University, Beni-Suef 62511, Egypt; mariam.marzouk@science.bsu.edu.eg (M.A.M.); manal.mohamed@science.bsu.edu.eg (M.M.Y.); salwashabaan@science.bsu.edu.eg (S.A.S.); hamada.abdelgawad@science.bsu.edu.eg (H.A.); 2 Integrated Molecular Plant Physiology Research (IMPRES), Department of Biology, University of Antwerp, 2020 Antwerp, Belgium; gerritts@uantwerpen.be

**Keywords:** endophytic fungi, *Pelargonium graveolens*, Cd-stress, redox status, detoxification, tissue quality, essential oil

## Abstract

Heavy metal contamination in soil is increasing rapidly due to increasing anthropogenic activities. Despite the importance of rose-scented geranium as a medicinal plant, little attention was paid to enhancing its productivity in heavy metal-polluted soil. In this regard, endophytes improve plant resistance to heavy metal toxicity and enhance its tissue quality. Here, the impact of the three endophytic fungi *Talaromyces versatilis* (E6651), *Emericella nidulan*s (E6658), and *Aspergillus niger* (E6657) on geranium growth, tolerance, and tissue quality under cadmium (Cd) stress was investigated. In contrast to *E*. *nidulans*, *T*. *versatilis* and *A*. *niger* enhanced geranium growth and the stimulatory effect was more pronounced under Cd-stress. The three endophytes significantly alleviated Cd accumulation and increased mineral content in geranium leaves. In addition, endophytic fungi successfully alleviated Cd-induced membrane damage and reinforced the antioxidant defenses in geranium leaves. Inoculation with endophytes stimulated all the antioxidant enzymes under Cd-stress, and the response was more obvious in the case of *T*. *versatilis* and *A*. *niger*. To reduce the toxicity of tissue-Cd levels, *T*. *versatilis* and *A*. *niger* upregulated the detoxification mechanisms; glutathione-S-transferase, phytochelatin, and metallothionein levels. Moreover, endophytic fungi improved the medicinal value and quality of geranium by increasing total antioxidant capacity (TAC), phenolic compound biosynthesis (phenylalanine ammonia-lyase), and vitamin content as well as the quantity and quality of essential oil, particularly under Cd-stress conditions. The variation in the mechanisms modulated by the different endophytic fungi was supported by Principal Component Analysis (PCA). Overall, this study provided fundamental insights into endophytes’ impact as a feasible strategy to mitigate the phytotoxicity hazards of Cd-stress in geranium and enhance its quality, based on the growth and biochemical investigations.

## 1. Introduction

Rose-scented geranium (*Pelargonium graveolens*) is an important medicinal aromatic plant. The plant is commonly utilized for the extraction of essential oil (geranium oil). Approximately, the essential oil is produced as 70–170 kg ha^−1^ geranium [1]. The price of geranium oil is expensive and reaches $120 kg^−1^ [2]. Egypt is in the second grade after China as a main producer and exporter of geranium oil, and the Egyptian geranium oil globally comes in the second grade after the Reunion Island oil, concerning quality [1]. In Egypt, the majority of geranium production is mainly in Beni-Suef governorate (about 7 miles south of Cairo) [3]. The rose-scented geranium plant was reported to have antibacterial, antifungal, anti-inflammatory, antioxidant, antidiabetic, and insecticidal properties [4]. The plant is used in the fragrance industry, cosmetic products, aromatherapy, and flavor industry, in addition to therapeutic uses [5]. Therefore, efforts should be made to enhance both the quantity and quality of geranium to meet the challenge in the marketing route.

Studies revealed that geranium essential oil is mainly constituted of monoterpenes which are prominent in the oil such as citronellol and geraniol (those are determinants of oil quality and impart a pleasing rosy fragrance), linalool and isomenthone, with their derivatives, and sesquiterpenes such as γ-cadinene, germacrene D, and caryophyllene oxide [2]. Each mono and sesquiterpenes category is produced by a different biosynthetic pathway. Monoterpenes are produced via the plastidic 2C-methylerythritol-4-phosphate (MEP) pathway, while the cytosolic enzymatic mevalonic acid (MVA) pathway produces sesquiterpenes [6], with cross-talk existing between the two pathways. These pathways are affected by biological and environmental factors such as heavy metal contamination [6]. For instance, the plant produces essential oils, as a group of secondary metabolites, in response to biotic and abiotic factors [7].

Heavy metal contamination of soil is one of the environmental problems affecting plants, animals, and human health. It is increasing rapidly nowadays due to increasing industrial pollution and anthropogenic activities [8]. Cadmium (Cd) is one of the toxic heavy metals, even at very low concentrations. For the medicinal plants used as home remedies, the safety limit recommended by WHO is 0.3 µg Cd g^−1^ plant tissue [9]. As geranium essential oil is extracted by hydrodistillation, there is no risk of the accumulated Cd to be transferred from the plant tissue to the extracted essential oil [10]. Nevertheless, the accumulated Cd inhibits plant growth and development and causes physiological disorders mostly due to the induced nutrient deficiency and oxidative stress [11]. Elevated Cd in soil may disturb plant mineral nutrition leading to an imbalance in plant macro and micronutrients such as P, K, Cu, Fe, and Zn [11,12]. The excessive generation of reactive oxygen species (ROS) is among the earliest biochemical consequences of Cd-stress. Cd induces the overgeneration of ROS via inhibiting electron transfer in chloroplasts and mitochondria [12] or inactivation of enzymes [13]. Disturbance of mineral uptake and translocation concurrently with the uptake of toxic Cd as well as generation of ROS, all result in growth inhibition, metabolic enzymes dysfunction, lipid peroxidation, and membrane damage [11,12]. However, a special situation was monitored on medicinal plants under heavy metal stress, as the effect of environmental factors on the biomass of herb is inversely proportional to their effect on essential oil [7]. Despite the abundant research attempted to characterize and reveal the responses of various medicinal plants to heavy metal stress [14], responses of rose-scented geranium to heavy metals, generally, and Cd, particularly were rarely studied.

To cope with the above-mentioned toxicity, the plant initiates some defense mechanisms such as decreasing uptake and translocation of Cd, cell wall adsorption, and chelation and compartmentation [15,16]. In addition, the plant stimulates its enzymatic and non-enzymatic antioxidant defenses to scavenge ROS and mitigate Cd-induced oxidative stress [13,17]. Secondary metabolites also accumulate in response to metal stress and contribute to defense-related signaling, metal chelation, and scavenging ROS [18]. All these defenses are cost-effective for the growth of the plant. Under these circumstances, it is an agricultural and economical challenge to produce both a high quantity and a high quality of geranium oil and herbs. In this regard, Cd significantly inhibited geranium fresh herb, while non-significantly affecting the essential oils [10].

In addition to the defense mechanisms modulated by the plant itself, interaction with rhizospheric and endophytic microorganisms also plays an essential role in enhancing plant tolerance to metal [16]. Endophytes are all microorganisms inhabiting plant tissues inter or intracellularly at any interval of their life cycle without causing apparent harm to their host [19]. Many endophytes were reported to support their host plants in their adaptation under environmental stresses such as salinity, high temperature, drought, and heavy metals [20]. Endophytes decrease heavy metal availability and reduce metal uptake by the plant while promoting the uptake of macro and micronutrients [11,21]. Moreover, endophytes can produce organic acids and plant hormones that boost plant growth and stimulate defenses in plants [11]. In this respect, inoculation of tomato with the endophytic fungal *Penicillium janthinellum* LK5 maintained plant growth, mitigated membrane damage, and upregulated the defense-related endogenous phytohormones under Al-stress [22]. Recently, *Aspergillus niger* and *Penicillium chrysosporium* were reported to inhibit Cd and Pb uptake and reduce lipid peroxidation in the inoculated *Vicia faba* plants [23].

Researchers have paid great attention to the interaction of medicinal and aromatic plants with their associated fungal endophytes. Since endophytic fungi were found to improve the bioactive chemical composition of medicinal plants as well as their growth and development under stressful and non-stressful conditions [24,25]. This improvement is achieved by triggering and promoting the secondary metabolites pathways, enhancing mineral nutrition, and regulating secondary metabolites-related gene expression. For instance, transcriptome analysis of *Cinnamomum longepaniculatum* seedlings inoculated with *Penicillium commune* 2J1 and *Neurospora crassa* 3J1 revealed the upregulation of genes involved in the essential oil monoterpene-biosynthetic pathway and plant hormone-signal transduction [26]. Additionally, inoculation of *Piriformospora indica* enhanced the antioxidant ability, P and K^+^, and essential oil composition in peppermint under salt stress [24]. Similar enhancement of the quantity as well as the quality of the essential oils of *Atractylodes lancea* [27], *Cinnamomum longepaniculatum* [25], fennel [28], and *Thymus vulgaris* [29] was also reported.

Despite the importance of geranium as a medicinal plant, little attention was paid to enhancing its productivity in heavy metal-polluted soil. Therefore, we (1) studied the response of geranium to Cd-stress, (2) evaluated the effect of endophytic fungi on geranium, as a medicinal aromatic plant, and (3) appreciated the potentiality of the endophytes to alleviate Cd-stress in geranium. We, herein, report for the first time the stimulation of growth, alleviation of Cd toxicity, and enhancement of the quality of rose-scented geranium by the inoculation of three endophytic fungi (*Talaromyces versatilis*, *Emericella nidulans*, and *Aspergillus niger*.

## 2. Materials and Methods

### 2.1. Experimental Design and Treatments

The experiment was carried out according to a completely randomized factorial design, from December to February. Pots (15 cm × 15 cm) were filled with one kg of garden soil. The soil was mixed with prewashed and dried sand as a 2:1 ratio. The soil properties were: 30.4% sand, 38.3% clay, 31.3% silt, pH 7.9, EC 2.45 dSm^−1^, OM 3.27%, nitrogen (N) 0.113, phosphorus (P) 0.061, potassium (K) 0.04, zinc (Zn) 5.0 mg kg^−1^ soil, and no fertilization practices were applied to the soil. Mixed soil was sterilized by autoclaving to eliminate microorganisms, however, it could also induce changes in the soil organic matter structure [30]. For endophyte treatment, the foot base without (for the non-inoculated plants) or with mycelial growth was applied. Four treatments of endophytes were applied as following: (1) non-inoculated (NI) soil; (2) soil was supplemented with *Talaromyces versatilis* (E6651) fungus; (3) soil was supplemented with *Emericella nidulans* (E6658) fungus, and (4) soil was supplemented with *Aspergillus niger* (E6657) fungus. All the tested fungi were isolated from rose-scented geranium (*Pelargonium graveolens* (L’Hér) Thunb.) and identified microscopically and molecularly in our previous study [31]. Two surface-sterilized cuttings were planted in each pot at about a 3-cm depth in the soil. Pots were kept in net house under natural conditions and irrigated with tap water (200 mL per pot) every two days. The temperature range was 19–20 °C for maximum and 10–11 °C for minimum, and the humidity range was 58–63%. All the plants treated or not treated with endophytic fungi were divided into two groups; normal conditions (NC) and Cd-stressed; seven pots for each treatment. After the full development of the third leaf, about 73 days after planting, Cd-stress was applied by watering pots regularly with 200 mL cadmium chloride (CdCl_2_.2.5H_2_O; 50 µM) solution; based on a preliminary experiment. Plants were harvested 17 days after applying stress. Fresh weight (FW) of leaves was recorded, and samples were kept at −20 °C for further biochemical analyses.

### 2.2. Minerals Assay

About 150 mg of the dry plant samples were digested in HNO_3_/H_2_O solution (5:1 *v*/*v*) in an oven [32]. Minerals (Cd, K, Ca, Mg, P, Na, Fe, Cu, Mn, and Zn) were determined by ICP-MS (Finnigan Element XR, Scientific, Bremen, Germany).

### 2.3. Oxidative Markers 

Hydrogen peroxide (H_2_O_2_) was extracted in 0.1% trichloracetic acid (TCA), and its content in the extract was estimated using 1 M potassium iodide (KI), following the protocol of Velikova et al. [33]. The concentration of H_2_O_2_ was expressed as mmol H_2_O_2_ g^−1^ FW. The level of lipid peroxidation in a tissue sample can be measured as the concentration of malondialdehyde (MDA), a principal product of lipid peroxidation [34], which reacts with thiobarbituric acid (TBA). The concentration of MDA was expressed as µmol MDA g^−1^ FW. 

### 2.4. Activity of Enzymatic Antioxidants

Enzymatic antioxidants were extracted from 200 mg of frozen plant material in 2 mL extraction buffer (50 mM potassium phosphate buffer, pH 7, contained 10% PVP, 0.25% Triton X-100, 1 mM PMSF and 1 mM ASC). After centrifugation for 10 min at 13,000 rpm and at 4 °C, the supernatants were used to spectrophotometrically evaluate the activities of superoxide dismutase (SOD, EC: 1.15.1.1), catalase (CAT, EC: 1.11.1.6), peroxidase (POX, EC: 1.11.1.7), ascorbate peroxidase (APX, EC: 1.11.1.11), monodehydroascorbate reductase (MDHAR, EC: 1.6.5.4), dehydroascorbate reductase (DHAR, EC: 1.8.5.1), glutathione reductase (GR, EC: 1.6.4.2) and glutathione peroxidase (GPX, EC: 1.11.1.9). The activity of SOD was determined as USOD mg^−1^ (protein) min^−1^ by monitoring the inhibition of nitroblue tetrazolium (NBT) reduction at 560 nm [35]. CAT activity was assayed by monitoring the breakdown of H_2_O_2_ at 240 nm and expressed as µmol H_2_O_2_ mg^−1^ (protein) min^−1^ [36]. The activity of POX was determined based on the oxidation of pyrogallol and expressed as µmol oxidized pyrogallol mg^−1^ (protein) min^−1^ [37]. Those of APX, MDHAR, DHAR, and GR were assayed as previously described [38] and expressed as µmol AsA mg^−1^ (protein) min^−1^, µmol NADH mg^−1^ (protein) min^−1^, µmol AsA mg^−1^ (protein) min^−1^ and µmol NADPH mg^−1^ (protein) min^−1^, respectively. The activity of GPX was assayed by measuring the decrease in NADPH absorbance at 340 nm and expressed as µmol NADPH mg^−1^ (protein) min^−1^ [39]. Protein content was estimated according to Lowry et al. [40].

### 2.5. Detoxification Related Parameters

Glutathione-S-transferase (GST, EC: 2.5.1.18) enzyme was extracted in 50 mM of potassium phosphate buffer at pH 7.0. The activity was measured spectrophotometrically following the resultant conjugation of reduced glutathione (GSH) and 1-chloro-2,4-dinitrobenzine (CDNB) and expressed as µmol GSH-CDNB conj. mg^−1^ (protein) min^−1^ [41]. Glutathione (GSH) was measured by HPLC methods. GSH was separated by using 2 mM KCl at pH 2.5 as elution buffer on a reverse-phase column (100 × 4.6 mm Polaris C_18_-A, 3 mm particle size; 40 °C). Total reduced GSH was determined after reducing with 0.04 M DTT. To measure total phytochelatins (PCs), total non-protein thiols in plant samples were extracted in 5% sulfosalicylic acid, mixed with Ellman’s reagent, and quantified spectrophotometrically at 412 nm [42]. The total phytochelatins content was estimated from the difference between the total non-protein thiol and total glutathione (GSH) content [43]. Electrochemically, metallothioneins (MTs) content was measured by using the differential pulse voltammetry Brdicka reaction [44].

### 2.6. Biological and Medicinal Value

#### 2.6.1. Total Antioxidant Capacity

The total antioxidant capacity (TAC) in geranium leaves was estimated by two chemical reactions. The first is a modified ferric ion reducing antioxidant power (FRAP) assay [45]. After extraction in 80% ethanol and centrifugation at 14,000 rpm for 20 min, the supernatant was mixed with acetate-buffer (0.3 M, pH 3.6), containing 10 mM 2,4,5-Tris-(2-pyridil)-s-triazine (TPTZ) and 200 mM FeCl_3_. The absorbance was measured at 600 nm and results were expressed as Trolox equivalents (µmol g^−1^ FW). The second chemical reaction measured the scavenging activity of the free radical 1,1-diphenyl-2-picrylhydrazyl (DPPH) in a methanolic extract. The absorbance was measured at 517 nm and antioxidant activity was expressed as % of scavenging.

#### 2.6.2. Phenylalanine Ammonia-Lyase (PAL) Activity and Flavonoids

The activity of phenylalanine ammonia-lyase (PAL, E.C: 4.3.1.5) was estimated by detecting the cinnamic acid formed as a secondary product from the enzyme reaction. The enzyme was extracted in 5 mL of 0.05 M borate buffer (pH 8.8) containing 1 mM ethylenediaminetetraacetic acid (EDTA), 2 mM β-mercaptoethanol and 4% (*w*/*v*) polyvinylpyrrolidone (PVP) to 200 mg plant fresh tissue. The mixture was homogenized then centrifuged at 10,000 rpm for 15 min at 4 °C. For estimation of PAL activity, 0.1 mL enzyme extract was added to 0.3 mL of 50 mM L-phenylalanine. The reaction was incubated at 30 °C for 15 min before adding 0.1 mL 6 N HCl to stop the enzymatic reaction. The mixture was read at 290 nm [46]. The activity of PAL was expressed as µmol trans-cinnamic acid g^−1^ FW h^−1^, and the concentration of cinnamic acid was calculated from a standard curve. For extraction of total flavonoids, plant samples were extracted in 80% methanol, and after centrifugation at 6000 rpm for 15 min at room temperature [47], the supernatant was used for the assay. Total flavonoid content was estimated according to the method described by Zhishen et al. [48] using NaNO_2_ and AlCl_3_. The absorbance was measured at 510 nm, and the concentration was calculated using the quercetin standard curve. The content of total flavonoids was expressed as mg quercetin g^−1^ dry wt. 

#### 2.6.3. Vitamins 

The content of carotenes (vitamin A) and phylloquinone (vitamin K) were determined by HPLC [49,50]. Carotenes were extracted in acetone and quantified by a reversed-phase HPLC (software analysis with Shimadzu Lab Solutions Lite). Phylloquinone was detected by reversed-phase HPLC (RP18 column, Eurosphos- 100, 250 × 4.6 mm, Germany) using a fluorescence detector (excitation, 243 nm; emission, 430 nm). Thiamine (vitamin B1) was estimated by the spectrofluorimetric method. The method is based on the oxidation of thiamine with ferricyanide to form fluorescent thiochrome [51]. Ascorbic acid (vitamin C) was measured by the HPLC method. Ascorbic acid was separated as mentioned above for GSH. Tocopherols were separated and quantified by HPLC (Shimadzu, ‘s-Hertogenbosch, The Netherlands) (normal phase conditions, Particil Pac 5 µm column material, length 250 mm, i.d. 4.6 mm) [52].

#### 2.6.4. Essential Oil Analysis 

The essential oil was extracted from plant samples by hydrodistillation using Clevenger-type apparatus. After extraction, the oil was collected and dried over anhydrous sodium sulfate. Oil content (%) and oil yield (mL plant^−1^) were calculated. The essential oil chemical constituents were analyzed by gas chromatography–mass spectrometry (GC-MS). Separation was performed using a gas chromatograph HP-5 (Crosslinked 5% PH ME Siloxane, 15 m × 0.53 mm × 1.5 μm film) column at helium flow rate 2 mL min^−1^, injector temperature 220 °C and detector temperature 240 °C using temperature program 60 °C, 40 °C min^−1^ up to 220 °C, 2 min at 220 °C. Portions of 2 μL of the essential oil (dissolved in hexane) were injected into the used analytical column. Identification of oil components was achieved based on their retention indices (RI, determined with reference to a homologous series of normal alkanes) and by comparison of their mass spectral fragmentation patterns (NIST) database (G1036A, revision D.01.00)/Chem-Station data system (G1701CA, version C.00.01.08).

### 2.7. Statistical Analysis

All the data were statistically analyzed using the SPSS program v.19. Two-way ANOVA was applied to study the effect of Cd-stress (Cd), endophytes (E), and their interaction on geranium. One-way ANOVA with Post Hoc-Tukey HSD test (*p* ≤ 0.05) was applied to assess the differences among means of treatments. Values were expressed as means of replicates ± SE. Principal component analysis (PCA) was generated by Multi Experimental Viewer (TM4 software package, MEV 4.7, http://mev.tm4.org, accessed on: 30 August 2021).

## 3. Results

### 3.1. Fungal Isolation and Identification 

The three endophytic fungi were isolated from different organs (leaves, stems, and roots) of the medicinal plant *Pelargonium graveolens* according to our previous article [31]. In more detail, the isolated fungi were purified on antibiotic-free PDA (Potato dextrose agar) media. Then DNA was extracted from a single spore of each fungal isolate and subjected to molecular identification where the nuclear DNA region containing the internal transcribed spacer 1 and 2 (ITS1 and ITS2) and two regions of the rRNA gene cluster, was amplified by PCR. ITS sequences of the isolates have been subjected to sequence similarity comparison of the sequences from the NCBI GenBank database (www.ncbi.nlm.nih.gov, accessed on: 30 August 2021).

### 3.2. T. versatilis and A. niger- Inoculation Enhanced Biomass Accumulation

**Figure 1:**
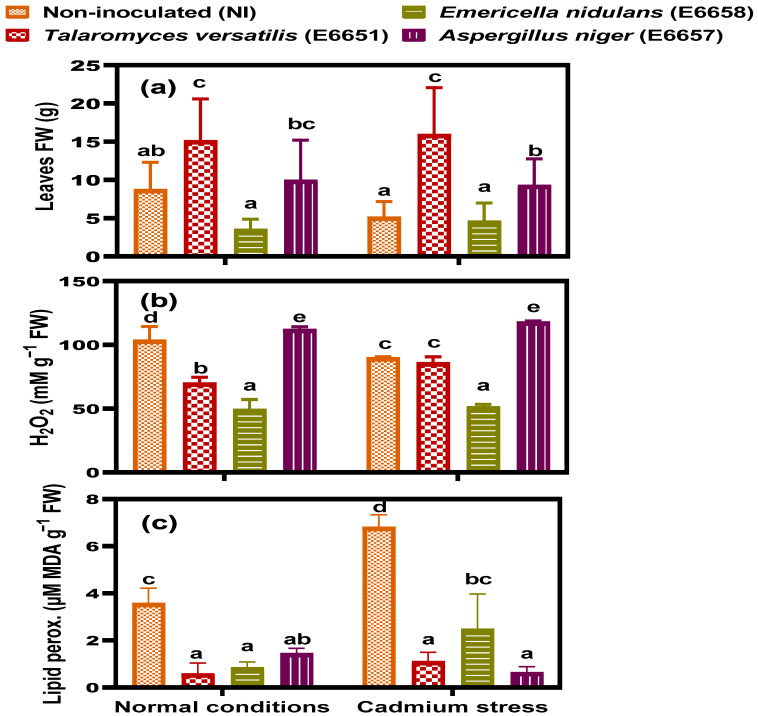
(**a**) Total leaves fresh weight, (**b**) content of H_2_O_2_ and (**c**) lipid peroxidation (MDA) of geranium non-inoculated (NI) or inoculated with different endophytic fungi; *T*. *versatilis* (E6651), *E*. *nidulans* (E6658) and *A*. *niger* (E6657) under normal conditions and Cd-stress; after 17 days of applying stress. Values are expressed as means ± SE (*n* = 5–8) for (**a**) and (*n* = 3) for (**b**,**c**). For comparison among different treatments within and between levels of both normal conditions and cadmium stress, values with at least one similar letter are non significantly different according to One-way ANOVA with Post Hoc-Tukey HSD test (*p* ≤ 0.05).

The effect of inoculating geranium with the fungal endophytes *T*. *versatilis* (E6651), *E*. *nidulans* (E6658), and *A*. *niger* (E6657) on biomass accumulation under Cd stress was investigated (Figure 1). Since fresh leaves are the main bulk of biomass yield and the main source of geranium oil extracted from the plant [53], we studied the growth in terms of the fresh weight of leaves. Under normal conditions, the endophyte *T*. *versatilis* followed by *A. niger* had the most stimulatory effect on geranium leaves fresh biomass (Figure 1), while *E*. *nidulans* showed the opposite effect. In this context, *T*. *versatilis* significantly increased biomass by 73% over the control, but *A*. *niger* only induced a non-significant increase of 14%. Under Cd-stress, the biomass of non-inoculated geranium decreased obviously to 58.4% of control. The stimulatory effect of *T*. *versatilis* and *A*. *niger* was more pronounced under stressful conditions, as they significantly stimulated the biomass of geranium leaves to 312% and 182% respectively, compared to the non-inoculated one under Cd-stress. In contrast, *E*. *nidulans* inhibited leaf growth.

### 3.3. Endophytic Fungi Alleviated Cd Accumulation and Enhanced Mineral Composition

In this study, we investigated the mineral content in geranium to understand the effect of Cd-stress and endophytic fungi on nutrient uptake. Moreover, it will help to appreciate the plant quality and how endophytes can entangle with the nutrient quality of the plant under stressful and non-stressful conditions. The influence of endophytes on the content of most geranium minerals was highly significant (*p* ≤ 0.0001) (Table S1). Applying Cd, however, did not induce a significant change, as the main effect, in all the estimated minerals, except K and Cd (*p* ≤ 0.05 and *p* ≤ 0.001 respectively).

Under normal conditions, Cd was not detected (Table 1), however applying Cd-stress increased its content in the leaves of the non-inoculated geranium. While leaves inoculated with *T*. *versatilis* showed a nonsignificant change in Cd accumulation compared to that of the non-inoculated ones, both *E*. *nidulans* and *A*. *niger* significantly alleviated Cd accumulation in geranium leaves. At optimum conditions, a significant increase in most of the investigated mineral contents was observed (Table 1), indicating an enhancement of the mineral nutrition in geranium. This increase was the most apparent in the case of inoculating with *T*. *versatilis* for Mg, P, Na, Mn, and Zn. However, *A*. *niger* was superior by scoring the highest K, Ca, and Mg content, in addition to elevating P, Na, Mn, and Zn over control (Table 1). In non-inoculated plants, applying stress sharply declined the mineral contents, particularly that of K, Ca, P, Mn, and Zn, compared with normal conditions. However, inoculating geranium with endophytes not only alleviated that decline but also augmented the level of most minerals over that of control due to the significant interaction (E × Cd; Table S1) effect. That response was the most apparent with the plants inoculated with *E*. *nidulans*. 

### 3.4. Endophytic Fungi Mitigated the Response of Geranium to Cd-Induced Oxidative Stress 

Heavy metal stress, along with other stresses, produces reactive oxygen species (ROS), including H_2_O_2_, that cause damage to plant’s protein, DNA, lipid molecules, and cell membranes. To evaluate the induced oxidative stress due to applying endophytes and Cd-stress on geranium, we estimated the contents of H_2_O_2_ and MDA (lipid peroxidation). While inoculation with the endophyte *E*. *nidulans* displayed the lowest level of H_2_O_2_, *A*. *niger* expressed the highest one under normal conditions, exceeding the control (Figure 1). Notably, H_2_O_2_ content decreased in the non-inoculated geranium leaves under Cd-stress. Due to the significant interaction effect (E × Cd; *p* ≤ 0.001; Table S1), the response of H_2_O_2_ in geranium leaves was strain-dependent. The content of H_2_O_2_ in *T*. *versatilis*-inoculated samples non-significantly differed from that of the non-inoculated ones under Cd-stress. While *E*. *nidulans* declined the level of H_2_O_2_ in geranium leaves, *A*. *niger* augmented that level under stressful conditions. Regarding cellular membrane damage, all the independent factors, endophytes (E) and Cd, and their interaction (E × Cd) significantly affected lipid peroxidation (*p* ≤ 0.001; Table S1). Inoculation with the three applied endophytes significantly attenuated the level of lipid peroxidation when compared with the non-inoculation under normal conditions (Figure 1). While Cd-stress alone intensified lipid peroxidation of geranium leaves, the combination of endophytes and Cd successfully alleviated the membrane damage, indicating the reinforcement of the antioxidant defense as a result of the inoculation with endophytic fungi. 

### 3.5. T. versatilis and A. niger- Inoculation Stimulated Antioxidant Defense under Cd-Stress

**Figure 2:**
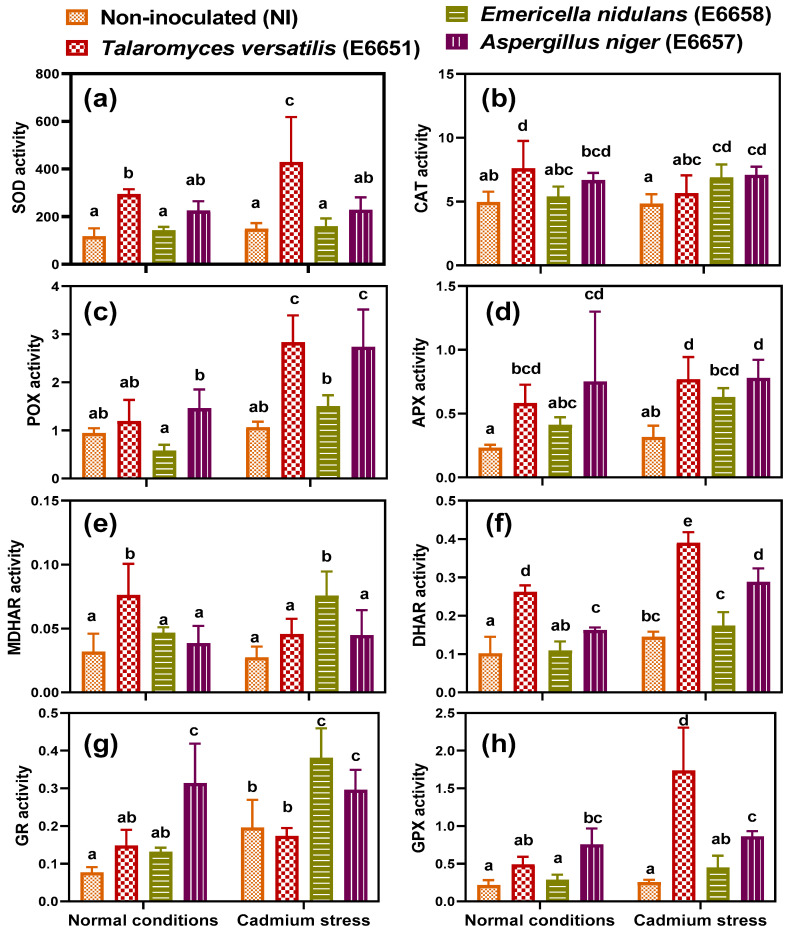
(**a**) Superoxide dismutase (SOD, USOD mg^−1^ (protein) min^−1^), (**b**) catalase (CAT, µmol H_2_O_2_ mg^−1^ (protein) min^−1^), (**c**) peroxidase (POX, µmol oxidized pyrogallol mg^−1^(protein) min^−1^), (**d**) ascorbate peroxidase (APX, µmol AsA mg^−1^(protein) min^−1^), (**e**) monodehydroascorbate reductase (MDHAR, µmol NADH mg^−1^(protein) min^−1^), (**f**) dehydroascorbate reductase (DHAR, µmol AsA mg^−1^ (protein) min^−1^), (**g**) glutathione reductase (GR, µmol NADPH mg^−1^ (protein) min^−1^) and (**h**) glutathione peroxidase (GPX, µmol NADPH mg^−1^ (protein) min^−1^) activities in leaves of geranium non-inoculated (NI) or inoculated with different endophytic fungi; *T*. *versatilis* (E6651), *E*. *nidulans* (E6658) and *A*. *niger* (E6657) under normal condition and Cd-stress; after 17 days of applying stress. Values are expressed as means ± SE (*n* = 4). For comparison among different treatments within and between levels of both normal conditions and cadmium stress, values with at least one similar letter are non significantly different according to One-way ANOVA with Post Hoc-Tukey HSD test (*p* ≤ 0.05).

To understand how the endophytes inoculation alleviated lipid peroxidation and membrane damage, we studied the enzymatic antioxidant defense. Antioxidant enzymes (SOD, POX, CAT, APX, MDHR, DHAR, GR, and GPX) play a major role in scavenging ROS and protecting cellular components against oxidative damage [54]. The activity of all the antioxidant enzymes changed significantly in response to the endophytes (*p* ≤ 0.0001 to *p* ≤ 0.01; Table S1). Consequently, under normal conditions, *T*. *versatilis* was able to stimulate SOD, CAT, APX, MDHAR, and DHAR in their host plants over the non-inoculated plants (Figure 2). The stimulation reached up to 2.5-fold of the control for APX, MDHR, and DHAR, indicating the ability of *T*. *versatilis* to increase the recycling of ascorbates even under normal conditions. Additionally, *A*. *niger* was found to increase the activity of CAT, APX, DHAR, GR, and GPX. In contrast, *E*. *nidulans* did not induce any significant change in the activity of antioxidant enzymes when compared with the control. After Cd-stress application, the non-inoculated plants showed increases in the activity of DHAR and GR compared with the control (Figure 2). As a result of Cd-stress, most of the investigated antioxidant enzymes responded differently to the respective fungal endophyte by the significant interaction effect (Cd × E; *p* ≤ 0.0001–*p* ≤ 0.05). Inoculation with the studied endophytes stimulated all the antioxidant enzymes under Cd-stress (Figure 2), indicating its effectiveness in reinforcing the antioxidant defense system. Generally, that response was more obvious in the case of *T*. *versatilis* and *A*. *niger* than with *E*. *nidulans*, despite inducing the highest stimulation of MDHAR and GR in the plants inoculated by the latter. 

### 3.6. Inoculation with Fungal Endophytes Reinforced Cd-Detoxification Mechanisms in Geranium

To reveal how geranium cope with the toxicity of Cd and the reinforcement of the detoxification mechanisms by endophytes, heavy metal detoxification mechanisms including phytochelatins (PCs), metallothioneins (MTs), and glutathione-S-transferase (GST) were studied. Both PCs and MTs are polypeptides involved in the chelation and detoxification of heavy metals [13,55].

**Figure 3:**
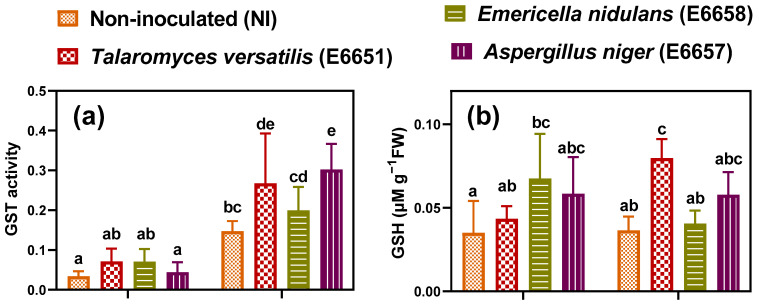

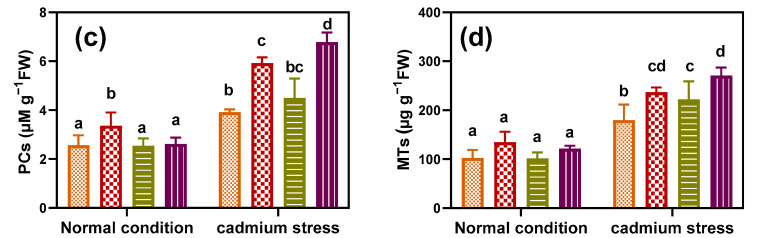
(**a**) Activity of glutathione S-transferase (GST, µmol GSH-CDNB conj. mg^−1^ (protein) min^−1^), (**b**) reduced glutathione (GSH), (**c**) phytochelatins (PCs) and (**d**) metallothioneins (MTs) in leaves of geranium non-inoculated (NI) or inoculated with different endophytic fungi; *T*. *versatilis* (E6651), *E*. *nidulans* (E6658) and *A*. *niger* (E6657) under normal conditions and Cd-stress; after 17 days of applying stress. Values are expressed as means ± SE (*n* = 3–4). For comparison among different treatments within and between levels of both normal conditions and cadmium stress, values with at least one similar letter are non significantly different according to One-way ANOVA with Post Hoc-Tukey HSD test (*p* ≤ 0.05).

GST has a role in the transport of GSH-metal and phytochelatin-metal complexes to the vacuole and the removal of toxic products of lipid peroxidation [55]. The activity of GST did not change significantly by the endophyte inoculation under non-stressful conditions (Figure 3a). In contrast, Cd-stress significantly augmented the activity of that enzyme in both the non-inoculated and inoculated geranium, regardless of the level of Cd accumulation. The highest activity was scored by *A*. *niger*, that increased GST activity by about 2-fold of that in the non-inoculated samples, followed by *T*. *versatilis*. We also estimated the content of GSH for its involvement in chelation with metals and being the structural unite of PCs [13]. Under optimum conditions, *T*. *versatilis* non significantly changed its content, while *E*. *nidulans* and *A*. *niger* significantly increased their content of GSH to a level higher than that of control (Figure 3b). Despite the non-significant change in GSH level of the non-inoculated geranium plants, plants inoculated with *T*. *versatilis* and *A*. *niger* significantly increased this level under Cd-stress (Figure 3b). We also estimated the content of PCs, in addition to metallothioneins (MTs), due to their implication in heavy metal chelation and sequestration, resulting in oxidative damage mitigation. Both PCs and MTs showed a similar response to GST, indicating their integrated work in Cd detoxification (Figure 3c,d). There was no significant change of PCs and MTs in most treatments under normal conditions, while their levels were markedly enhanced in both the inoculated and non-inoculated geranium under Cd-stress. However, the level in the inoculated leaves was higher revealing the boosting role of endophytes in the detoxification mechanisms in geranium against Cd. The endophyte *A*. *niger* followed by *T*. *versatilis* was the most efficient in increasing PCs and MTs in geranium under Cd-stress (Figure 3).

### 3.7. Biological and Medicinal Value

#### 3.7.1. Inoculation with Endophytes Enhanced Total Antioxidant Capacity (TAC) and Stimulated Phenolic Metabolism

**Figure 4:**
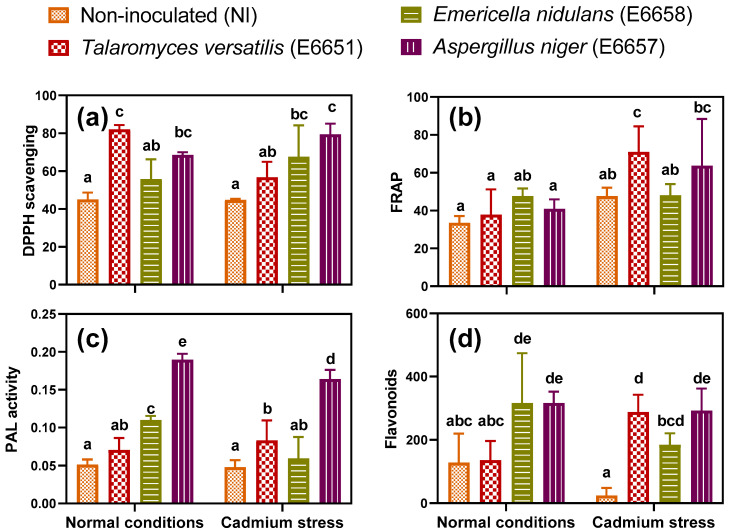
(**a**) The scavenging activity of the free radical 1,1-diphenyl-2-picrylhydrazyl (DPPH scavenging, %), (**b**) ferric ion reducing antioxidant power (FRAP, µmol g^−^^1^ FW), (**c**) phenylalanine ammonia-lyase (PAL, µmol trans-cinnamic acid g^−^^1^ FW h^−^^1^) activity and (**d**) flavonoids (mg quercetin g^−1^ dry wt) content of leaves of geranium non-inoculated (NI) or inoculated with different endophytic fungi; *T*. *versatilis* (E6651), *E*. *nidulans* (E6658) and *A*. *niger* (E6657) under normal conditions and Cd-stress; after 17 days of applying stress. Values are expressed as means ± SE (*n* = 3–4). For comparison among different treatments within and between levels of both normal conditions and cadmium stress, values with at least one similar letter are non significantly different according to One-way ANOVA with Post Hoc-Tukey HSD test (*p* ≤ 0.05).

We used two different chemical reactions, the Ferric Reducing Ability of Plasma (FRAP) and DPPH scavenging activity, to evaluate the total antioxidant capacity (TAC). For FRAP, the inoculated plants did not significantly differ from the non-inoculated plants at optimum conditions (Figure 4). Applying Cd-stress, however, significantly enhanced this capacity, particularly in plants inoculated with *T*. *versatilis* and *A*. *niger* relative to the corresponding control (Figure 4). Concerning DPPH, both *T*. *versatilis* and *A*. *niger* inoculated plants showed an enhancement in their antioxidant capacity over control (NI) under normal conditions (Figure 4). Applying Cd-stress did not induce a significant difference in the scavenging activity of the non-inoculated plants. In contrast, all the endophyte-inoculated plants showed an increment of their scavenging activity under Cd-stress, indicating that endophytes armed their hosts well with the antioxidants appropriate to face Cd-induced oxidative stress. There was a significant interaction (Cd × E; *p* ≤ 0.05) between stress (Cd) and endophytes (E), reflecting how the effect of the applied endophytes on antioxidants varied across the different stressful conditions.

We assayed the enzyme phenylalanine ammonia-lyase (PAL) as the first enzyme in the phenylpropanoid pathway that is involved in the biosynthesis of many phytoceutical compounds produced by geranium, including flavonoids and some essential oil traces. Therefore, evaluating PAL activity in geranium will allow a better understanding of the basis that underlies the endophytes induced high biological and medicinal potentiality. In the current study, all the inoculated endophytes were able to enhance their host PAL activity under normal and Cd-stress conditions, indicating the ability of endophytic fungi to trigger the phenylpropanoid pathway. This stimulation was the highest with *A*. *niger* (Figure 4). Flavonoid contents were significantly changed by the effect of endophytes (*p* ≤ 0.001; Table S1) or their interaction with Cd stress (E × Cd; *p* ≤ 0.05). Under non-stressful conditions, leaves developed from the *E*. *nidulans* and *A*. *niger* inoculated plants showed a significant increase of flavonoids content compared with control (Figure 4). While Cd alone obviously decreased flavonoids concentration, below control, all the inoculated plants significantly augmented this content in response to Cd-stress. The endophyte *A*. *niger* was the most effective in scoring the highest flavonoids concentration in geranium leaves under both stressful and non-stressful conditions.

#### 3.7.2. Inoculation with Endophytic Fungi Increased Biological and Medicinal Value of Cd-Stressed Geranium Plants

As geranium is an aromatic and medicinal plant, we estimated the vitamins and essential oil contents to study the inoculation effect on the medicinal value of Cd-stressed plants. In this regard, we measured vitamins, oil content (%), and essential oil yield, and we also analyzed the chemical constituents of geranium oil and calculated the citronellol/geraniol (C/G) ratio to reveal the changes in the quantity and quality of the essential oil. Our data revealed that the different estimated vitamins, particularly vitamin A, B, and K, showed similar patterns of change in response to most of the different treatments (Table 2). Under normal conditions, while *T*. *versatilis* did not induce a significant change in the content of most vitamins of geranium, *A*. *niger* increased the content of these vitamins, particularly vitamin A (α-carotene and β-carotene) and vitamin E (tocopherols). Although *E*. *nidulans* dramatically reduced the level of vitamins A, B (thiamine), and K (phylloquinone), it induced accumulation of vitamins C (ascorbic acid) and E (alpha tocopherol) in the inoculated geranium relative to control. However, those responses differed significantly by applying Cd stress, and the interaction effect (E × Cd) was significant (*p* ≤ 0.0001; Table S1) for all the tested vitamins. All the investigated endophytes significantly stimulated the production of vitamins in their host under Cd-stress compared with control (Table 2), and the highest stimulation was shown by *A*. *niger* followed by *T*. *versatilis*. Among the vitamins that increased in response to endophyte inoculation under Cd-stress, were vitamin A (β-cryptoxanthin, α-carotene, and β-carotene), thiamine, and phylloquinone. Only, the plants inoculated with *A*. *niger* showed a significant accumulation of vitamin C in response to Cd-stress (Table 2). Tocopherols (vitamin E) were obviously increased in response to Cd pollution, but the highest levels were scored by *A*. *niger* followed by *T*. *versatilis*. 

Essential oils belong to different chemical classes, including monoterpenoids, sesquiterpenoids, and phenylpropanoids [6]. Studies revealed that geranium essential oil is mainly constituted of citronellol, linalool, geraniol, and isomenthone, belonging to monoterpenes, in addition to sesquiterpenes [2]. Since the production of essential oils and their quality is affected by environmental conditions, the response of the evaluated compounds was dependent on the endophytes and plantation conditions. The analysis in our study revealed the identification of eleven of the essential oil constituents, and the major components were citronellol and geraniol (Table 2). Two-way ANOVA analyses revealed that endophytes significantly affected the oil content, oil yield, C/G ratio, and all the estimated constituents of the oil (Table S1). At optimum conditions, *A*. *niger* induced a significant enhancement to the oil content, oil yield, and most of the essential oil chemical constituents including citronellol and geraniol. Although *T*. *versatilis* and *E*. *nidulans* had no significant effect on the citronellol and geraniol contents, they improved some of the other constituents belonging to monoterpene esters and sesquiterpene hydrocarbons that found in the essential oil as traces. While *T*. *versatilis* did not affect the percentage of oil content or C/G ratio significantly, *E*. *nidulans* enhanced the quality of the essential oil by inducing a C/G ratio of almost one (1.04), but notably decreased both oil yield and leaves biomass, indicating mostly that its effect was on the quality rather than quantity. Cd alone had no significant effect on the essential oil constituents; thus, it did not affect significantly neither the quality nor the quantity of the essential oil in the non-inoculated plants. The three chemical constituents, citronellol, trans-geraniol, and geraniol, together with caryophyllene oxide showed a significant response to the interaction effect of endophytes and Cd-stress (E × Cd; Table S1) ranged from (*p* ≤ 0.01) to (*p* ≤ 0.001). Under Cd-stress, the studied endophytic fungi increased the content of most of the essential oil constituents in leaves of the inoculated plants, and the effect of *A*. *niger* was superior by inducing the maximum improvement of the chemical constituents. *E*. *nidulans* and then *T*. *versatilis* induced less effect, particularly for citronellol and geraniol (Table 2). While the abundance of monoterpenes (35.5%) in the essential oil of non-inoculated geranium decreased to about 30%, inoculation with *A*. *niger* efficiently increased this abundance to 62.4%. Moreover, *A*. *niger* was the most effective in increasing the oil content by 130% over the control in the Cd-stressed geranium leaves. Besides, it increased the concentration of citronellol and geraniole to 1.58 and 2-fold of the control respectively, inducing a C/G ratio (1.33) to approach one as an indication of the enhanced quality of geranium essential oil under Cd-stress.

### 3.8. Principal Component Analysis (PCA) Revealed the Variation in the Mechanisms Modulated by the Different Endophytic Fungi

**Figure 5:**
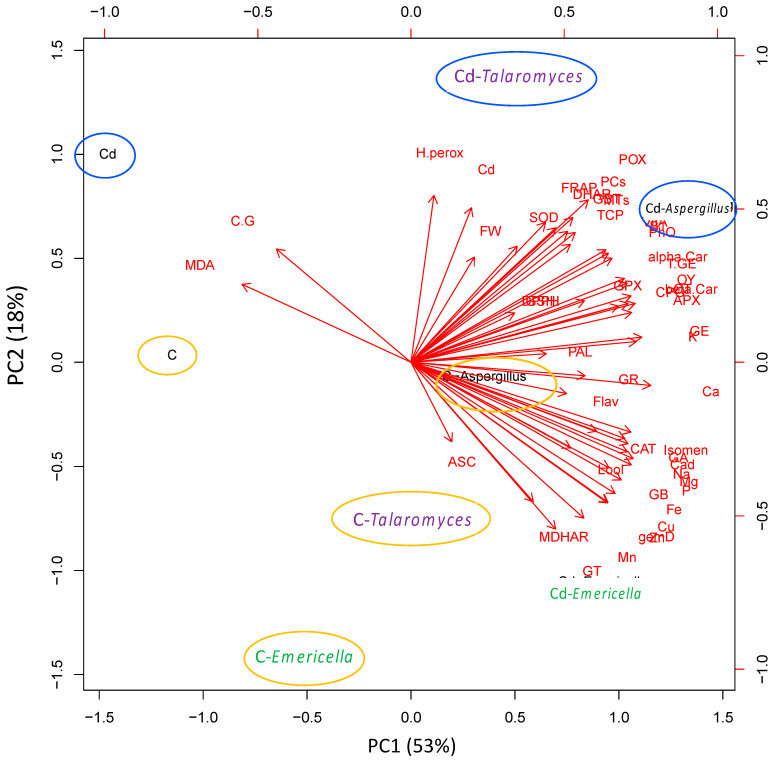
Principle component analysis (PCA) of growth and biochemical parameters of geranium leaves as affected by the inoculation with different endophytic fungi; *T*. *versatilis* (E6651), *E*. *nidulans* (E6658), and *A*. *niger* (E6657) under normal conditions and Cd-stress. C; non-inoculated geranium under normal conditions, C-*Talaromyces*, C-*Emericella* and C-*Aspergillus;* geranium inoculated with different endophytic fungi under normal conditions, Cd; non-inoculated geranium under Cd-stress, Cd-*Talaromyces*, Cd-*Emericella* and Cd-*Aspergillus;* geranium inoculated with different endophytic fungi under Cd-stress, FW; leaves fresh weight, H perox; hydrogen peroxide, MDA; lipid peroxidation, SOD; superoxide dismutase, CAT; catalase, POX; peroxidase, APX; ascorbate peroxidase, MDHAR; monodehydroascorbate reductase, DHAR; dehydroascorbate reductase, GR; glutathione reductase, GPX, glutathione peroxidase, DPPH; 1,1-diphenyl-2-picrylhydrazyl, FRAP; ferric ion reducing antioxidant power, PAL; phenylalanine ammonia-lyase, Flav; flavonoids, GST; glutathione S-transferase, GSH; glutathione, PCs; phytochelatins, MTs, metallothioneins, VitA; β-cryptoxanthin, alpha-Car; α-carotene, beta-Car; β-carotene, B1; thiamine, PhQ; phylloquinone, ASC; ascorbic acid, TCP; tocopherols, CT; citronellol, T.GE; trans-geraniol, Isomen; isomenthone, Lool; linalool, GA; geranylacetate, Cad; cadinene, GB; geranyl butyrate, GT; geranyl tiglate, gemD; gemacreneD, CPO; caryophyllene oxide, GE; geraniol, C.G; citronellol/geraniol ratio, OY, oil yield.

To test the specific responses of geranium plants to the different endophytic fungi under Cd-stress, we performed the principal component analysis (PCA) to get a global overview and to understand the basis of this variation (Figure 5). The PCA embodied uniform growth and biochemical parameters along the first two dimensions (PC1 and PC2) that represented 53% and 18% of the data variability, respectively. PC1 separated the measured parameters on the basis of inoculation impact, particularly under Cd stress (53% of all data variables).

The change in MDA level explained this separation, where the different endophytic fungi reduced MDA value clearly, particularly with Cd stress increase. For PCA2, which accounts for 18% of responses variation, the separation was Cd-stress-isolate-dependent. While the measured parameters for *T*. *versatilis* and *A*. *niger* showed similar trends, there was a distinct variation in *E*. *nidulans* responses, under both normal and stressful conditions. The separation of *E*. *nidulans* under normal conditions was due to its distinct enhancement of the redox homeostasis. Under Cd-stress, the separation of *E*. *nidulans* was due to its stimulatory effect on most mineral contents and fine constituents of essential oils. In contrast, *T*. *versatilis* grouped at the opposite side was explained by the increase in fresh weight, Cd accumulation, and antioxidant defense (SOD, POX, DHAR, and FRAP). *A*. *niger* separated in a group between *E*. *nidulans* and *T*. *versatilis* along PC2 by enhancing most vitamin contents, and by sharing some medicinal value parameters (essential oil constituents, citronellol, and geraniol) with the former and detoxification responses (PCs, MTs and GST) with the later.

## 4. Discussion

### 4.1. Inoculated Geranium Plants Showed Improved Growth and Mineral Nutrition under Cd-Stress 

Increased Cd in the soil inhibits the growth and mineral composition of plants [56]. It also alters plant tissue chemical composition that can negatively affect tissue quality. Endophytes, on the other hand, have been recognized as a revolutionary approach to improve their host plant growth, chemical composition, and resistance to environmental stresses. Here we found that both *T*. *versatilis and A*. *niger* enhanced geranium leaves biomass under both normal conditions and Cd-stress. There was a declined Cd content in the plants that inoculated with *E*. *nidulans* and *A*. *niger*. The effect of endophytes on geranium biomass was in an isolate-dependent manner. The difference in the tendency to accumulate Cd between plants inoculated by *T*. *versatilis* and those inoculated by *A*. *niger*, despite increasing biomass of both, could be related to the ability of the fungi themselves to accumulate Cd [56]. For instance, it was reported that the Cd biosorption of *A*. *niger* is higher than that of *Penicillium chrysosporium*, and the higher ability of the former to bio-accumulate and bind Cd to its cell wall, leads to the decrease of Cd-uptake by plant and reduce its availability in the soil [23]. 

The decrease in Cd accumulation was also associated with enhanced mineral nutrition by enhancing the geranium content of some essential macro and microelements such as K, Ca, Mg, P, Fe, Cu, Mn, and Zn. This enhancement is presumably due to the increased mobilization of those minerals as a result of the inoculation with the endophytes. Minerals are widely involved in structural and functional roles in plants [57]. The role of endophytes in improving the mineral composition of their host and even the ability to moderate heavy metal stress by enhancing minerals uptake was documented [58]. Increasing the plant content of all these minerals, particularly Ca that compete with Cd for the same Ca^2+^ channels, was found to enhance Cd tolerance [17]. However, the inoculated plant that accumulated more Cd, in the current study, showed a higher increase in biomass, indicating that the enhanced growth due to inoculation with specific endophytes could be attributed to other reasons rather than the enhanced mineral nutrition. That reveals again the difference in the mechanisms triggered by each to enhance Cd-tolerance in geraniums. That view was supported by the PCA (Figure 5).

### 4.2. Inoculated Geranium Plants Were Less Sensitive to Cd Oxidative Stress

It is well known that overaccumulation of H_2_O_2_ induces damage to cell membranes and macromolecules [59]. In comparison with the non-inoculated plants, those inoculated with *E*. *nidulans* and *T*. *versatilis* declined H_2_O_2_ concentration in the different applied conditions, indicating the enhancement in the H_2_O_2_ scavenging system or the decrease in its generation. In contrast, *A*. *niger* induced the highest H_2_O_2_ concentration. Notably, this accumulation was not accompanied by oxidative damage, as indicated by lipid peroxidation results, suggesting a probability of utilizing H_2_O_2_ as a signaling molecule [25,27]. In line with our findings, heavy metal stress mitigation was correlated with a declined lipid peroxidation under inoculation with fungal endophytes such as *Penicillium funiculosum* LHL06 and *Penicillium janthinellum* LK5 (PJLK5) [22,60].

The plant developed many strategies to scavenge the excessive ROS and prevent their accumulation. These strategies employ enzymatic antioxidants, such as SOD, CAT, POX, APX, MDHAR, DHAR, GR, and GPX, and non-enzymatic antioxidants such as glutathione, ascorbic acid, and flavonoids [13,54,59]. Endophytes were extensively studied regarding this issue and were found to stimulate many mechanisms that help plants not only to survive but also to grow healthy under stress conditions [61,62,63]. Endophytes were suggested to produce ROS that works as signals to stimulate plant antioxidant enzymes and affect its ROS balance “acquired immune system” [64], explaining the increment of H_2_O_2_ in *A*. *niger*–inoculated geranium under both normal conditions and Cd-stress (Figure 1). In our study, *T*. *versatilis* and *A*. *niger*, in most cases, had a stimulating effect on the antioxidant enzymes of geranium plants under control conditions and to more extent under Cd stress. It was found that the inoculation of the endophytic fungal strain; *Alternaria alternata* RSF-6L alleviated oxidative stress through increasing antioxidant enzymes activity of *Solanum nigrum* [56]. Moreover, the endophyte *Penicillium janthinellum* LK5 improved aluminum phytoextraction in *Solanum lycopersicum* and mitigated Al-oxidative stress by up-regulating antioxidants and endogenous salicylic acid [22]. Recently, we reported an implication of SOD and antioxidant defenses in Cd tolerance [65] Overall, the endophytic fungal inoculation augmented the antioxidant activity of geraniums. This matches the growth results and explains how these endophytes alleviated oxidative stress (H_2_O_2_ and lipid peroxidation) in geraniums. 

### 4.3. Improved Cd Detoxification Level Can also Explain Endophytes-Induced Tolerance in Geranium

Chelation of metal and compartmentation in vacuoles and other organelles are among the detoxification mechanisms of Cd [17]. Chelation is accomplished through the binding of the metal to PCs which are synthesized from their precursor GSH by the enzyme phytochelatin synthase [66], or through binding metal directly to GSH [67]. Plants also chelate Cd with MTs which are cysteine-rich proteins [13]. In the current study, the endophyte inoculation did not significantly affect the response of GST, PCs, and MTs, while augmented GSH levels relative to control under normal conditions. The increased GSH levels in the inoculated plants under normal conditions could be related to the involvement of GSH in the redox homeostasis [68] that is probably managed by the endophytes. GSH can scavenge H_2_O_2_, singlet oxygen (^1^O_2_), hydroxyl radical (OH^•^), and superoxides (O_2_^•−^). Besides its role in signaling, GSH reacts with the lipid peroxidation metabolites and gives resistance against the product of lipid peroxidation (MDA) [13]. Exposure of geranium to Cd significantly stimulated GST in both the inoculated and non-inoculated leaves. The enzyme GST is vital in the conjugation of GSH and thiol compounds to heavy metals and its higher expression confer tolerance against Cd-stress [69]. Cd also induced a significant accumulation in both PCs and MTs in all the investigated samples, but the response of PCs and MTs, similar to GST, was significantly higher with *T*. *versatilis* and *A*. *niger* compared to the non-inoculated plants. These responses linked with that of GSH indicate that both *T*. *versatilis* and *A*. *niger*, specifically, boosted the detoxification mechanisms and granted geranium protection against the accumulated Cd toxic ions. We, herein, report for the first time the adoption of these detoxification strategies by the investigated endophytes, particularly *T*. *versatilis* and *A*. *niger*. The highest levels of GSH in the *T*. *versatilis* inoculated plants may explain its highest biomass despite the high accumulation of Cd. Since tolerance and detoxification of Cd were reported to depend on the ability of the plant to maintain high levels of GSH instead of PCs [13,70]. Similar induction of GSH was reported on *Penicillium janthinellum* LK5 (PJLK5) in its host tomato plant [22] and *Penicillium funiculosum* LHL06 in its host soybean plant [60] against Al and Cu toxicity, respectively. The nonsignificant effect of *E*. *nidulans* on most of the above-mentioned detoxification strategies confirms their cruciality in Cd-tolerance and interprets the decline of biomass of its host plant. 

### 4.4. Endophyte Increased Medicinal and Economic Values of Geranium Plant by Improving Its Tissue Chemical Composition and Quality

Geranium is an important essential oil plant, thus its constituents of vitamins and essential oils determine its phytoceutical values, as well as its activity against oxidative stress. Therefore, it is important to investigate the endophyte impact on heavy metal-induced alteration in geranium tissue quality and bioactivity. Plants inoculated by *T*. *versatilis* and *A*. *niger* induced the highest total antioxidant capacity (TAC) under normal conditions as well as Cd-stress. Similar results were reported by El-Mahdy et al. [23], who found that the endophytes *Aspergillus niger* and *Penicillium chrysosporium* enhanced the antioxidant capacity of faba bean under Cd-stress. In the same context, the endophytic fungus *Aspergillus* sp. isolated from *Lippia citriodora* was reported to increase its host antioxidant capacity [71]. 

Activation of phenylpropanoid pathway as a result of stimulating the PAL enzyme to produce many phenolic compounds is one of the mechanisms plants accelerate under heavy metal stress [18]. Flavonoids are known to have high antioxidant activity [47]. In addition to their role in stress mitigation, flavonoids can improve plant biological activity [18]. In our study, all the isolates, particularly *A*. *niger* were able to increase PAL activity that was linked, in most cases with the accumulated flavonoids. Similarly, fungal inoculation of *Epichloë* sp. and *Thermomyces lanuginosus* endophytes to *Achnatherum sibiricum* [72] and *Cullen plicata* [73] plants enhanced the activity of PAL enzyme, accumulated total phenolics, and flavonoids, and increased the tolerance of the infected plants to insect herbivores and heat stress respectively. 

The quality of geranium is mainly determined based on its essential oil content and its composition. Geranium essential oil is mainly constituted of monoterpenes (citronellol, geraniol, linalool, and isomenthone), and sesquiterpenes (γ-cadinene, germacrene D, and caryophyllene oxide) [2]. In our study, *A*. *niger* was the most effective endophyte in increasing the oil content and enhancing the percentage of chemical constituents in essential oil, particularly the main ones; citronellol and geraniol, under optimum conditions. Although *E*. *nidulans* enhanced the C/G ratio to approach one and hence increased the essential oil quality, it non-significantly affected the quantity. In response to Cd toxicity that declined geraniol concentration and decreased oil content and composition [10], *A*. *niger*, followed by *E*. *nidulans*, effectively enhanced both the quality and quantity of geranium oil, by increasing the concentration of most of the essential oil constituents, particularly geraniol, balancing the C/G ratio and increasing percentage of oil content and oil yield, which prospectively leads to increased economic and medicinal values. Moreover, this enhancement could be linked with high mineral uptake and mobilization. In this context, mineral nutrition is one of the factors regulating the biosynthesis of essential oils [7]. For instance, sufficient uptake of the inorganic P is required for isoprenoid biosynthesis (the building unite of essential oils) [74], and enhancing the divalent metal ions, such as Mg^2+^ or Mn^2 +^, facilitates functioning as a catalyst for the enzymes synthesizing monoterpenes [6,74]. In our study, the stimulated mineral uptake shown by the plants inoculated with both *A*. *niger* and *E*. *nidulans*, while inhibited in the non-inoculated ones under Cd-stress justifies the enhanced oil content and composition. 

Being the main constituents in geranium oil, the change in the levels of citronellol and geraniol accounts for the major differences among the essential oils of geranium inoculated with the different three endophytes. At essential oil composition, it was reported that geraniol and citronellol are highly negatively correlated, geraniol is the precursor of citronellol [7]. In *Pelargonium*, geraniol can be transformed in a sequential pathway catalyzed by different enzymes into geranial, geranyl acetate, and geranyl formats in one hand, or into citronellol by a direct reduction or through a multi-step pathway involving both alcohol dehydrogenase and reductase enzymes in another hand [75]. Although *T*. *versatilis*, induced the least enhancement in the essential oil constituents, it scored the highest quantity (oil yield), indicating that the improvement of its host biomass could compensate for the nonsignificant effect on the quality, confirming that the effect on the biomass of geranium is inversely proportional to that on essential oil [7].

For the vitamins that are essential not only for humans but also for plants, their contents were also altered in Cd-stressed plants. In their review, Asensi-Fabado and Munne’-Bosch [76] reported that all the plant derived-vitamins have antioxidant activity. They are powerful antioxidants, play an important role in redox chemistry, and are cofactors as well [76]. The pro-vitamin A, a lipid-soluble vitamin, can scavenge ^1^O_2_ [68]. Thiamine, a water-soluble vitamin has a scavenging affinity to O_2_^•−^ and OH^•^, whereas phylloquinone, an essential component of photosynthetic electron transport, is involved in redox reactions in plasma membranes [76]. Tocopherols react with lipid peroxy radicals and maintain membranes integrity by reducing lipid peroxidation [76]. None of the above-mentioned vitamins was modulated properly by the non-inoculated plants. In contrast, all the inoculated plants significantly increased their contents of vitamins, pertaining to the declined lipid peroxidation, enhanced TAC, and promoted antioxidant defense. The enhanced vitamins of the inoculated plants confirm the ability of endophytes to increase the biological and medicinal value of geranium, and *A*. *niger* accomplished these effects efficiently by increasing the content of all the investigated vitamins. The increased vitamins in response to endophytes could come via their effect on metabolism, including hormone biosynthesis pathway that shared with that of most vitamins [76], or by triggering specific defense mechanisms in geraniums.

## 5. Conclusions

Understanding the role of endophytic fungi in induced responsiveness geranium plants to Cd-stress is worthwhile. Endophytic fungi, particularly *T*. *versatilis* and *A*. *niger* mitigated Cd stress impact on geranium growth. They enhanced geranium biomass as well as its tissue chemical composition and quality. Under Cd-stress, the inoculated fungi enhanced the content of most geranium essential oil constituents, and *A*. *niger* was the most effective in increasing the oil content, quality, and yield in Cd-stressed geranium leaves. Cd uptake was reduced by endophytes inoculation. This was accompanied by improving the detoxification system; PCs, MTs and GST. Endophytic fungi increased the resistance to Cd-induced oxidative changes through the enhancement of the antioxidant defense system. Our findings also showed a different pattern in geranium responses to different endophytic fungi under Cd-stress. This variation was further supported by Principal Component Analysis (PCA). For further understanding of the signaling and molecular mechanisms of endophytic positive impact on plant responses to Cd-stress, underlying detailed metabolomics, and transcriptomic studies are needed.

## Figures and Tables

**Table 1:** Mineral content in leaves of geranium non-inoculated (NI) or inoculated with different endophytic fungi under normal conditions and Cd-stress; after 17 days of applying stress.

Minerals	Normal Conditions				Cadmium Stress			
	NI	*T. versatilis* (E6651)	*E. nidulans* (E6658)	*A. niger* (E6657)	NI	*T. versatilis* (E6651)	*E. nidulans* (E6658)	*A. niger* (E6657)
Cd	0.000 ± 0.00 a	0.00 ± 0.00 a	0.00 ± 0.00 a	0.00 ± 0.00 a	8.351 ± 0.51 c	8.187 ± 0.62 c	6.586 ± 0.64 b	6.352 ± 0.84 b
K	1.440 ± 0.09 bc	1.36 ± 0.24 bc	1.14 ± 0.20 ab	1.94 ± 0.19 d	0.780 ± 0.05 a	1.85 ± 0.12 cd	2.070 ± 0.19 d	2.270 ± 0.10 d
Ca	0.028 ± 0.00 b	0.035 ± 0.01 bc	0.03 ± 0.00 bc	0.04 ± 0.00 cd	0.017 ± 0.00 a	0.04 ± 0.00 cd	0.047 ± 0.00 d	0.051 ± 0.00 d
Mg	0.410 ± 0.02 ab	0.61 ± 0.11 cd	0.54 ± 0.06 bc	0.62 ± 0.05 cd	0.252 ± 0.03 a	0.53 ± 0.05 bc	0.850 ± 0.04 e	0.768 ± 0.09 de
P	2.840 ± 0.23 b	4.12 ± 0.59 cd	3.71 ± 0.31 bc	4.09 ± 0.34 cd	1.550 ± 0.11 a	3.58 ± 0.26 bc	5.370 ± 0.35 d	4.950 ± 0.54 de
Na	0.378 ± 0.01 a	0.73 ± 0.14 bc	0.71 ± 0.08 bc	0.68 ± 0.05 bc	0.261 ± 0.03 a	0.606 ± 0.07 b	0.75 ± 0.06 bc	0.876 ± 0.09 c
Fe	0.060 ± 0.01 ab	0.08 ± 0.0 bcd	0.08 ± 0.0 bcd	0.07 ± 0.01 bc	0.039 ± 0.00 a	0.067 ± 0.01 b	0.103 ± 0.01 d	0.098 ± 0.01 cd
Cu	0.010 ± 0.00 ab	0.02 ± 0.0 bcd	0.02 ± 0.0 bcd	0.016 ± 0.0 bc	0.008 ± 0.00 a	0.014 ± 0.0 bc	0.022 ± 0.00 d	0.019 ± 0.00 cd
Mn	0.034 ± 0.00 b	0.06 ± 0.01 cd	0.053 ± 0.0 cd	0.052 ± 0.00 c	0.016 ± 0.00 a	0.05 ± 0.01 bc	0.069 ± 0.00 d	0.046 ± 0.00 bc
Zn	0.060 ± 0.01 b	0.09 ± 0.02 c	0.090 ± 0.01 c	0.089 ± 0.01 c	0.030 ± 0.00 a	0.08 ± 0.01 bc	0.120 ± 0.01 d	0.095 ± 0.00 cd

Values are expressed as means ±SE (*n* = 4). Values with at least one similar letter are non significantly different for each element (for each row) according to One-way ANOVA with the Post Hoc-Tukey HSD test (*p* ≤ 0.05). The elements’ concentration was expressed as mg g^−1^ FW, except Cd, Cu, Mn, and Zn were expressed as µg g^−1^ FW.

**Table 2:** Vitamin content, essential oil chemical constituents (%), oil content (%), and oil yield (mL Plant^−1^) of geranium leaves in response to inoculation with endophytic fungi under normal conditions and Cd-stress; after 17 days of applying stress.

	Normal Conditions				Cadmium Stress			
	NI	*T*. *versatilis* (E6651)	*E*. *nidulans* (E6658)	*A*. *niger* (E6657)	NI	*T*. *versatilis* (E6651)	*E*. *nidulans* (E6658)	*A*. *niger* (E6657)
*Vitamins*								
β-Cryptoxanthin (Vit. A)	1.27 ± 0.07 bc	1.27 ± 0.09 bc	0.73 ± 0.06 a	1.48 ± 0.09 cd	1.05 ± 0.09 b	1.61 ± 0.14 d	1.56 ± 0.09 cd	1.94 ± 0.11 e
α-Carotene (Vit A)	0.79 ± 0.05 bc	0.89 ± 0.07 cd	0.54 ± 0.03 a	0.99 ± 0.05 de	0.68 ± 0.05 ab	1.03 ± 0.05 e	1.06 ± 0.04 de	1.26 ± 0.06 f
β-Carotene (Vit. A)	0.54 ± 0.01 b	0.67 ± 0.01 c	0.43 ± 0.01 a	0.71 ± 0.01 cd	0.48 ± 0.00 ab	0.72 ± 0.01 cd	0.78 ± 0.01 d	0.90 ± 0.01 e
Thiamine (Vit B)	1.10 ± 0.06 bc	1.11 ± 0.08 bc	0.64 ± 0.05 a	1.29 ± 0.07 cd	0.91 ± 0.07 b	1.39 ± 0.11 d	1.36 ± 0.07 d	1.68 ± 0.09 e
Ascorbic acid (Vit. C)	0.18 ± 0.07 a	0.17 ± 0.01 a	0.27 ± 0.03 c	0.17 ± 0.01 a	0.19 ± 0.00 a	0.17 ± 0.01 a	0.21 ± 0.00 ab	0.25 ± 0.01 bc
Tocopherols (Vit. E)	31.9 ± 0.89 a	58.1 ± 11.1 ab	48.3 ± 8.3 ab	63.7 ± 12.2 b	56.4 ± 1.3 ab	66.1 ± 11.7 b	48.6 ± 4.2 ab	77.4 ± 14.1 b
Phylloquinone (Vit. K)	0.92 ± 0.05 bc	0.95 ± 0.07 bc	0.56 ± 0.04 a	1.09 ± 0.06 cd	0.77 ± 0.06 b	1.17 ± 0.09 d	1.16 ± 0.06 d	1.42 ± 0.07 e
*Essential oil (%)*								
Citronellol (*1*.*216*)	17.4 ± 1.0 abc	16.5 ± 3.0 ab	13.7 ± 2.0 a	23.5 ± 2.0 cd	14.8 ± 1.00 a	22.0 ± 2 bcd	25.3 ± 2.0 d	27.6 ± 1.0 d
Trans-geraniol (*1*.*166*)	2.60 ± 0.2 ab	2.4 ± 0.4 a	2.20 ± 0.3 a	3.40 ± 0.3 bc	2.10 ± 0.10 a	3.60 ± 0.2 c	3.50 ± 0.2 c	3.90 ± 0.5 c
Isomenthone (*1*.*144*)	1.30 ± 0.2 a	2.4 ± 0.6 bcd	2.3 ± 0.3 abcd	2.40 ± 0.2 bcd	1.60 ± 0.2 ab	2.10 ± 0.3 abc	3.00 ± 0.2 cd	3.20 ± 0.3 d
Linalool (*1*.*082*)	0.96 ± 0.1 ab	0.91 ± 0.1 ab	0.70 ± 0.04 ab	1.00 ± 0.1 b	0.60 ± 0.04 a	0.90 ± 0.1 ab	1.70 ± 0.2 c	1.10 ± 0.2 b
Geranyl acetate (*1*.*362*)	1.00 ± 0.04 a	2.0 ± 0.4 bc	1.90 ± 0.2 bc	1.90 ± 0.2 bc	1.00 ± 0.10 a	1.60 ± 0.2 ab	2.00 ± 0.2 bc	2.30 ± 0.2 c
γ-cadinene (*1*.*514*)	0.04 ± 0.01 a	0.08 ± 0.02 bc	0.07 ± 0.01 bc	0.08 ± 0.01 bc	0.05 ± 0.01 ab	0.06 ± 0.01 ab	0.1 ± 0.01 c	0.10 ± 0.01 c
Geranyl butyrate (*1*.*561*)	0.69 ± 0.10 a	0.92 ± 0.2 abc	0.91 ± 0.07 abc	0.86 ± 0.07 ab	0.70 ± 0.06 a	0.77 ± 0.07 a	1.20 ± 0.07 c	1.10 ± 0.2 bc
Geranyl tiglate (*1*.*572*)	0.10 ± 0.006 ab	0.18 ± 0.04 cd	0.17 ± 0.02 cd	0.17 ± 0.01 cd	0.07 ± 0.005 a	0.15 ± 0.02 bc	0.21 ± 0.02 d	0.13 ± 0.02 bc
Gemacrene D (*1*.*477*)	0.08 ± 0.01 ab	0.129 ± 0.02 c	0.125 ± 0.01 c	0.12 ± 0.01 c	0.07 ± 0.00 a	0.11 ± 0.01 bc	0.17 ± 0.01 d	0.132 ± 0.01 cd
Caryophyllene oxide (*1*.*583*)	1.80 ± 0.06 a	1.60 ± 0.3 a	2.20 ± 0.3 ab	2.90 ± 0.1 bc	1.70 ± 0.2 a	2.80 ± 0.2 bc	2.70 ± 0.3 bc	3.30 ± 0.4 c
Geraniol (*1*.*246*)	11.4 ± 1.1 a	11.4 ± 1.8 a	13.0 ± 1.7 ab	17.1 ± 0.6 c	9.20 ± 1.0 a	16.6 ± 1.0 bc	18.9 ± 1.1 c	23.1 ± 1.2 d
C/G	1.53 ± 0.1 bc	1.43 ± 0.1 bc	1.04 ± 0.1 a	1.37 ± 0.9 abc	1.66 ± 0.2 c	1.36 ± 0.2 abc	1.33 ± 0.1 abc	1.20 ± 0.1 ab
Oil content (%)	0.31 ± 0.02 a	0.39 ± 0.03 ab	0.34 ± 0.05 a	0.49 ± 0.03 bc	0.39 ± 0.03 ab	0.53 ± 0.02 cd	0.62 ± 0.03 de	0.73 ± 0.07 e
Oil yield (mL Plant^−1^)	27.3 ± 2.2 b	59.5 ± 4.8 d	12.1 ± 1.7 a	48.9 ± 2.8 c	20.3 ± 1.6 ab	84.9 ± 3.2 e	28.5 ± 1.4 b	67.7 ± 6.1 d

The bracketed italicized values indicate the retention index (RI). Values are expressed as means ± SE (*n* = 4). Values with at least one similar letter are non significantly different for each parameter (for each row) according to One-way ANOVA with the Post Hoc-Tukey HSD test (*p* ≤ 0.05). NI; non-inoculated, C/G; citronellol:geraniol ratio.

## Data Availability

Data of this study are included in the article or Supplementary Materials.

## Supplementary Materials

The following are available online at https://www.mdpi.com/article/10.3390/jof7121039/s1, Table S1. Two-way ANOVA (df, F-value and probability) for geranium total leaves fresh weight and biochemical analyses as affected by endophytic fungi inoculation (E), cadmium stress (Cd) and their interaction (E × Cd) effect.
