# Supplementary material for: Harnessing Endophytic Fungi for Enhancing Growth, Tolerance and Quality of Rose-Scented Geranium (Pelargonium graveolens (L’Hér) Thunb.) Plants under Cadmium Stress: A Biochemical Study
Source: J Fungi (Basel). 2021 Dec 3;7(12):1039. doi: 10.3390/jof7121039 (PMC8705862; doi:10.3390/jof7121039)
Supplement: Supplementary file 1 [file jof-07-01039-s001.zip › jof-1468243-supplementary/Supplementary material - Table S1.pdf]

Table S1. Two-way ANOVA (df, F-value and probability) for geranium total leaves fresh weight and biochemical analyses as affected by endophytic fungi inoculation (E), cadmium stress (Cd) and their interaction (E× Cd) effect.

Source	*df*	*leaves fresh weight*	*Cd*	*K*	*Ca*	*Mg*	*P*	*Na*	*Fe*
E	3	17.54****	2.5	12.9****	15.2****	15.7****	17.6****	14.8****	9.9***
Cd	1	0.308	494.7***	5.9*	3.2	1.7	0.42	0.003	0.38
E× Cd	3	1.003	2.5	8.8***	4.6*	6.1**	6.4**	2.1	3.8*
Error		(44)	(24)	(24)	(24)	(24)	(24)	(24)	(24)

Source	*df*	*Cu*	*Mn*	*Zn*	*H_2O_2*	*Lipid peroxidation*	*SOD*	*POX*	*CAT*
E	3	11.1****	16.5****	17.6****	157.8****	55.17****	15,86***	16.72****	4.803**
Cd	1	0.05	1.2	0.08	1.320	17.57***	3.156	45.84****	0.0090
E× Cd	3	3.8*	3.7*	4.3*	7.941**	9.419***	1.309	4.936**	3.179*
Error		(24)	(24)	(24)	(16)	(16)	(24)	(24)	(24)

Source	*df*	*APX*	*MDHAR*	*DHAR*	*GR*	*GPX*	*Antioxid-ant activity*	*FRAP*	*PAL activity*
E	3	7.605***	7.742***	87.81****	14.44****	24.96****	15.59****	0.665	69.10****
Cd	1	2.751	2.75	83.70****	20.23***	22.91****	0.05	13.47**	6.336*
E× Cd	3	0.3232	4.954**	4.655*	7.969***	12.54****	7.16**	2.129	4.282*
Error		(24)	(24)	(24)	(24)	(24)	(16)	(16)	(16)

Source	*df*	*Flavonoid-s*	*GST*	*GSH*	*Phytochel-atins*	*MTs*	*β-Cryptoxanthin (Vit A)*	*Thiamine (Vit B)*	*Phylloquinone (Vit K)*
E	3	9.390***	3.058*	3.087	18.46****	8.103**	16.4****	17.4****	18.8****
Cd	1	0.7232	72.39****	0.1544	204.9****	168.7****	28.7****	29.6****	31****
E× Cd	3	4.051*	2.659	3.941*	11.83***	3.060	10.93***	11.4****	12****
Error		(16)	(16)	(16)	(16)	(16)	(24)	(24)	(24)

source	*df*	*α-Carotene (Vit A)*	*β-Carotene (Vit A)*	*ASC (Vit C)*	*Tocopherols*	*citronellol*	*trans-geraniol*	*Isomenthon-e*	*linalool*
E	3	24.5****	28.3****	7.608**	3.395*	8.848***	6.053**	6.837**	12.80****
Cd	1	33.8****	30.6****	0.6345	3.1	10.39**	12.12**	4.677*	0.0997
E× Cd	3	13.9****	13.9***	7.357**	0.594	2.759	3.282*	1.194	6.648**
Error		(24)	(24)	(16)	(16)	(24)	(24)	(24)	(24)

source	*df*	*Geranylac-etate*	*cadinene*	*geranyl butyrate*	*Geranyl-tiglate*	*Gemacren-e D*	*caryophyll-ene oxide*	*Geraniol*	*C/G%*
E	3	6.25**	8.26***	6.885**	5.081**	9.540***	10.30***	22.49****	4.71*
Cd	1	9.265**	3.219	0.371	1.319	2.278	10.55**	19.30***	0.28
E× Cd	3	1.967	1.392	0.973	9.241***	5.107**	2.606	2.877	1.56
Error		(24)	(24)	(24)	(24)	(24)	(24)	(24)	(24)

source	*df*	*Oil yield(%)*	*Total oil/fresh weight*

E	3	15.29****	115.6****
Cd	1	47.93****	31.67****
E× *Cd*	3	2.57	8.79***
Error		(24)	(24)
